# Experimental Investigations on Sustainable Dual-Biomass-Based Composite Phase Change Materials for Energy-Efficient Building Applications

**DOI:** 10.3390/ma18153632

**Published:** 2025-08-01

**Authors:** Zhiwei Sun, Wei Wen, Jiayu Wu, Jingjing Shao, Wei Cai, Xiaodong Wen, Chaoen Li, Haijin Guo, Yin Tang, Meng Wang, Dongjing Liu, Yang He

**Affiliations:** 1 School of Civil and Transportation Engineering, Ningbo University of Technology, Ningbo 315016, Chinajingjing.shao@nbut.edu.cn (J.S.);; 2 School of Mechanical Engineering Tongji University, Tongji University, Shanghai 201804, China; 3 School of Energy and Power Engineering, Jiangsu University, Zhenjiang 212013, China; 4 College of Environmental Science and Engineering, Donghua University, Shanghai 201620, China

**Keywords:** biomass-based phase change materials, building energy saving, reed straw, waste cooking fat

## Abstract

The incorporation of phase change material (PCM) can enhance wall thermal performance and indoor thermal comfort, but practical applications still face challenges related to high costs and potential leakage issues. In this study, a novel dual-biomass-based shape-stabilized PCM (Bio-SSPCM) was proposed, wherein waste cooking fat and waste reed straw were, respectively, incorporated as the PCM substance and supporting material. The waste fat (lard) consisted of both saturated and unsaturated fatty acid glycerides, exhibiting a melting point about 21.2–41.1 °C and a melting enthalpy value of 40 J/g. Reed straw was carbonized to form a sustainable porous biochar supporting matrix, which was used for the vacuum adsorption of waste fat. The results demonstrate that the as-prepared dual-Bio-SSPCM exhibited excellent thermal performance, characterized by a latent heat capacity of 25.4 J/g. With the addition of 4 wt% of expanded graphite (EG), the thermal conductivity of the composite PCM reached 1.132 W/(m·K), which was 5.4 times higher than that of the primary lard. The thermal properties of the Bio-SSPCM were characterized using an analog T-history method. The results demonstrated that the dual-Bio-SSPCM exhibited exceptional and rapid heat storage and exothermic capabilities. The dual-Bio-SSPCM, prepared from waste cooking fat and reed straw, can be considered as environmentally friendly construction material for energy storage in line with the principles of the circular economy.

## 1. Introduction

Carbon emissions from the entire building construction process in China account for over 50% of the country’s total carbon emissions, making the continuous and stable reduction in construction-related emissions a crucial factor in achieving China’s carbon neutrality goal [1,2]. Given the ongoing implementation of China’s rural revitalization strategy, it is highly significant to utilize new materials and technologies to enhance the quality of rural housing construction and improve indoor thermal environments in village dwellings, thereby enhancing the living comfort among rural residents. Using phase change material (PCM) in the envelope structure is an effective means to improve wall thermal performance and enhance indoor thermal comfort [3,4]. However, practical applications still face challenges such as high costs, low thermal conductivity [5], leakage issues, and flammability concerns [6].

Biomass, such as fatty acids derived from animal fat [7] or vegetable fats like soybean oil [8], exhibits significant potential in serving as exceptional and sustainable PCMs. Boussaba et al. [8] successfully synthesized a novel bio-based phase change material (PCM) by combining hydrogenated palm kernel vegetable fat with cellulose fibers. This composite PCM exhibits a melting point of 27.33 °C and a high latent heat capacity of 40.3 J/g, making it suitable for various building applications. The thermo-physical and chemical properties of three tree fruit oils, namely Allanblackia, shea butter, and palm kernel oil, were assessed by Lawer-Yolar et al. [9] Their findings revealed that Allanblackia oil exhibited a high latent heat of energy (80.53 J/g) and demonstrated the greatest potential for utilization as a phase change material (PCM) in thermal energy storage systems. Alipour et al. [10] proposed a novel composite PCM consisting of a mixture of methyl palmitate (MP) and decanoic acid (DA), both derived from vegetable oil. The results indicate that the eutectic binary mixture, comprising 30% MP and 70% DA, exhibits desirable properties as a PCM for thermal energy storage in building materials due to its high latent heat value of 202.3 J/g.

Biomass serves not only as a versatile working material but also as an abundant and sustainable carbon precursor for the encapsulation of PCM [11,12,13]. Prabhu et al. [14] discovered that various biomass materials derived from plants or animals possess the potential to serve as porous carbon donors, offering a cost-effective alternative to more expensive carbon particles such as CNT, CNF, and EG. The exceptional rheological and thermal properties of these materials have been specifically engineered for their application in the development of innovative and highly efficient form-stabilized PCM. Yun et al. [15] utilized calcium carbonate-based starfish as a microporous supporting material for encapsulating paraffin-based PCM. The resulting composite PCM exhibited a phase change temperature range of 22–28 °C and achieved a maximum latent heat capacity of 57.66 J/g, demonstrating its potential as an environmentally friendly heat storage material. Zhu et al. [16] proposed a novel bio-based phase change material (PCM) by incorporating discarded corn stem with polyethylene glycol, pyrrole, and multi-walled carbon nanotubes to form a composite PCM for photo-thermal conversion. Dasiewicz et al. [17] prepared MDF boards with varying PCM addition ratios (0%, 5%, 10%, 30%, and 50%) and evaluated their properties. The results indicated that increasing the PCM content enhanced thermal performance and reduced thickness expansion, although it led to a decline in mechanical properties. Ultimately, a PCM addition ratio of 28% was determined to be optimal, offering the best balance among all evaluated properties. This study aims to develop thermally active MDF boards suitable for furniture and interior design applications. Das et al. [18] revealed that softwood exhibits a superior porous structure, boasting a surface area of 441 m^2^/g in comparison to wheat straw. This characteristic renders it more suitable for PCM encapsulation. In our previous study [12], we prepared rice husk silica (RHS) and a rice husk carbon (RHC) supporting matrix from rice husk for n-octadecane encapsulation. The adsorption capacity of *n*-octadecane was significantly high for RHS and RHC, with relative enthalpies reaching up to 106.5 J/g and 116.3 J/g for n-octadecane/RHS and n-octadecane/RHC, respectively. However, currently, there are limited studies utilizing biomass as both a working substance and carrier in composite phase change materials.

In terms of environmentally friendly processes, waste cooking fats derived from cooked meat could serve as suitable candidates for natural-based and eco-friendly PCM. Fabiani et al. [19] investigated the potential of utilizing fatty pig and chicken parts derived from slaughterhouse residues as PCM. The proposed material exhibited excellent compatibility with the selected passive application and demonstrated promising thermal performance. The application of this material, however, was hindered by its low thermal conductivity and excessive supercooling [20]. Fortunately, biochar derivatives possess a highly porous structure, chemically stable properties, substantial pore capacity, robust ion exchange capability, exceptional thermal stability, and a significant specific surface area. These attributes effectively address the aforementioned issues.

To address the persistent challenges of high cost, leakage, and poor thermal performance in conventional phase change materials (PCMs), this study introduces a significant contribution by developing a novel, sustainable, dual-biomass-based shape-stabilized PCM (Bio-SSPCM). This work pioneers a circular economy approach by uniquely integrating two distinct waste streams: waste cooking fat (lard) serves as the latent heat storage medium, while waste reed straw is converted into a porous biochar (RSB) support matrix. The core contribution lies in demonstrating how this biochar matrix performs a dual function: its hierarchical porous structure not only provides robust shape stability to prevent lard leakage through capillary and Van der Waals forces but also acts as an effective nucleating agent, significantly mitigating the inherent supercooling issue found in fatty acids. Furthermore, this research systematically overcomes the critical limitation of low thermal conductivity by incorporating expanded graphite (EG), which establishes continuous thermal pathways within the composite, enhancing the thermal conductivity by 5.4 times compared to pure lard. Therefore, the novelty of this work is not merely the proposal of a new material but the creation of a multi-functional composite that simultaneously resolves the key issues of leakage, supercooling, and thermal conductivity by synergistically utilizing waste-derived biomass, offering a practical and economically viable solution for energy efficiency in buildings.

## 2. Materials and Methods

### 2.1. Materials

The reed straw powders were sourced from Ningbo, located in Zhejiang province, China. Expanded graphite (worm-like, 400 × expansion) was obtained from Suqian Nakate new material Technology Co., Ltd. (Suqian, China).

### 2.2. Preparation of Waste Fat-Based PCM from Animal Residues

**Figure 1:**
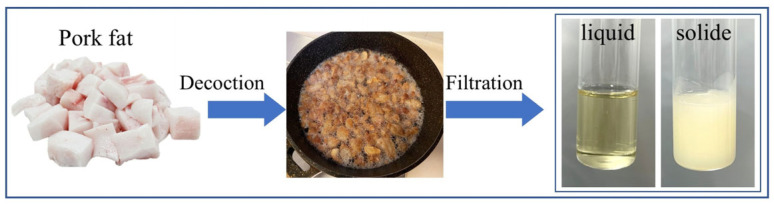
Preparation diagram of fat-based PCM.

The present study utilized pork fat as the source of animal residues for extracting waste fat. The pork fat, weighing 500 g, was thoroughly rinsed 2–3 times with deionized water to remove surface blood and dirt. Subsequently, the surface moisture was absorbed using filter paper, and the lard was cut into uniform 1 × 1 × 1 cm^3^ cubes. We placed the pork fat in a pot containing 100 mL of water and allow it to decoct for 30 min until complete rendering of fat occurs. Then, we separated any solid residue, collected the rendered lard, and transferred it into a dry beaker. The lard in molten and solidified states is depicted in Figure 1.

### 2.3. Preparation of Biomass Based Supporting Matrix and Composite PCM

**Figure 2:**
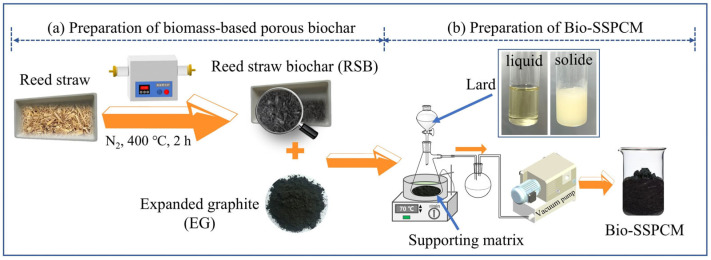
(**a**) Diagram of preparation of reed straw biochar; (**b**) diagram of preparation of Bio-SSPCM.

The reed straw-based biochar was prepared through high-temperature pyrolysis. The dried reed straw was placed in a quartz boat and subjected to pyrolysis in a tube furnace for 1 h at 600 °C under a N_2_ environment with a flow rate of 8 m/s. The high temperature carbonization process led to the production of reed straw biochar (RSB) at elevated temperatures. The preparation steps for biochar-based powders are depicted in Figure 2a.

The dual-biomass-based shape-stabilized PCM (Bio-SSPCM) was prepared using the molten vacuum impregnation method, as illustrated in Figure 2b. The supporting matrix, consisting of RSB and expanded graphite (EG), was positioned within a flask that was connected to a vacuum pump. Subsequently, the liquefied lard was introduced into the supporting matrix, followed by the release of vacuum and air intake to facilitate the infiltration of liquid lard into the same supporting matrix. The Bio-SSPCM was finally obtained, and the preparation process is displayed in Figure 2b. Table 1 presents the composition of different substances in the prepared composite PCMs. The leakage test was conducted to determine the maximum adsorption capacity of lard. Briefly, the molded samples were placed on filter paper and subsequently positioned on a heating plate (70 °C) to assess any potential lard leakage.

### 2.4. Characterization of Lard and Bio-SSPCM

The composition of extracted lard was separated and identified by gas chromatography–mass spectrometry (GC-MS,7890A-5975C, Agilent Technologies, Santa Clara, CA, USA) and infrared spectrum analysis (FT-IR, Nicolet iZ10, Thermo Scientific, Waltham, MA, USA, resolution 4.0 cm^−1^). The viscosity was measured using a rheometer (HAAKE MARS 60, Thermo Scientific, Waltham, MA, USA) for frequency scanning with a scanning range of 0.01–100 Hz and a fixed temperature of 25 °C. The pore structure and specific surface area of the biochar supporting matrix were assessed through nitrogen adsorption–desorption isotherm measurements using ASAP2020 (Micromeritics, Norcross, GA, USA). The microstructure and surface morphology of the supporting matrix and the Bio-SSPCM were examined using Field Emission Scanning Electron Microscopy (SEM, EM-30+, COXEM, Daejeon, Republic of Korea). The phase transition temperature, latent heat, and thermal reliability of the Bio-SSPCM were determined using a differential scanning calorimeter (DSC, Q2000, TA Instruments, New Castle, DE, USA) with a heating rate of 5 °C/min, with a temperature range of 0 °C to 80 °C. The thermal stability of the Bio-SSPCM was evaluated using a thermogravimetric analyzer (TG-DSC, STA 449, NETZSCH, Selb, Germany). The thermal conductivity of PCMs with stable shapes was tested by transient planar heat source method (Hot Disk TPS2500S, Gothenburg, Sweden).

## 3. Results and Discussion

### 3.1. Characterization of Waste Fat-Based PCM

**Figure 3:**
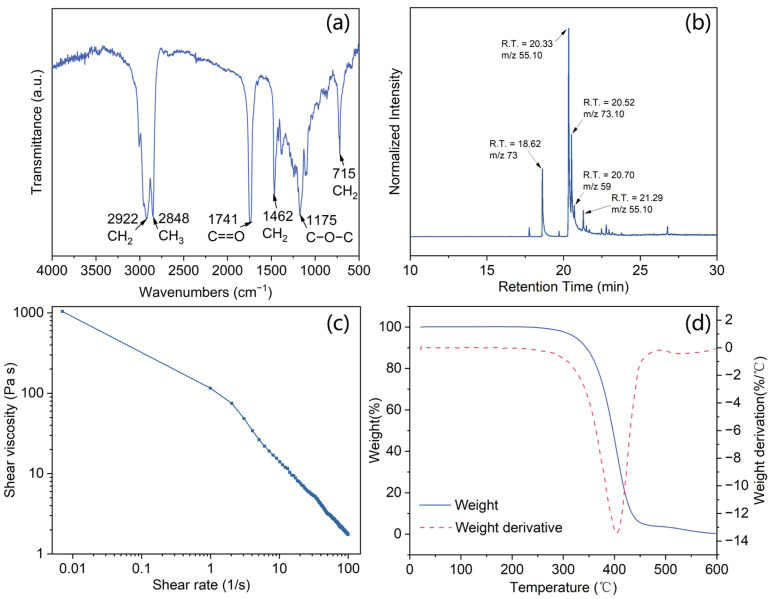
(**a**) FT-IR spectra; (**b**) GC-MS analysis; (**c**) frequency-sweep viscosity analysis; (**d**) thermogravimetric analysis of lard.

Figure 3a presented the FT-IR spectra of the waste fat-based PCM. The infrared spectrum indicates its affiliation with the fatty acid group, including oleic acid, linoleic acid, stearic acid, and palmitic acid. The peaks at 2920 and 2848 cm^−1^ correspond to the symmetric and asymmetric stretching of -CH_2_ and -CH_3_ groups, respectively. The peak at 1741 cm^−1^ is associated with the stretching of the carboxylic group C=O. Additionally, the peaks observed at 1462 cm^−1^ and 715 cm^−1^ can be attributed to CH_2_ bending and wagging, respectively. Finally, the presence of peaks at 1175 cm^−1^ signifies stretching vibrations of the C-O-C bond.

The GC-MS chromatogram depicted in Figure 3b facilitates the identification of fatty acids and enables a quantitative determination of their composition by comparing the eluted peak area with the cumulative sum of all peaks. The lard extracted from pork contains four types of fatty acids: 38.03% oleic acid (C18: 1, with an 18-carbon main chain and one internal unsaturation; *m*/*z* = 55.10; and a retention time (RT) of 20.33 min in Figure 3b), 12.73% palmitic acid (C16: 0; *m*/*z* = 73 and RT = 18.62 min), 21.49% stearic acid (C18: 0; *m*/*z* = 73.10 and RT = 20.52 min), and 12.09% hexadecanamide (C18: 2; *m*/*z* = 59 and RT = 20.70 min). The fatty acids account for 80% of the material, while the remaining 20% can be attributed to a small proportion of other fatty acids [20]. Additionally, it can be observed that the proportion of unsaturated fatty acids in lard is twice as high as that of saturated fatty acids. Typically, unsaturated fatty acids possess a liquid state at room temperature due to their low melting point, whereas saturated fatty acids exhibit a solid state at room temperature and have a higher melting temperature. The lard mixture thus demonstrates a gel-like structural composition.

The viscosity of the lard as a function of frequency is illustrated in Figure 3c. The shear viscosity decreases with an increasing shear rate, indicating a shear thinning behavior. Lard, composed of fatty acids and/or triglycerides, can be regarded as a complex network of entangled aliphatic chains. With the increase in the shear rate, chain alignment occurs, resulting in reduced interlayer friction [21]. Thermogravimetric analysis of the waste fat is displayed in Figure 3d. It shows one weight-loss step at nearly 400 °C is associated with the thermal degradation of the fatty acids and triglycerides, which implies that the lard has excellent thermal stability.

**Figure 4:**
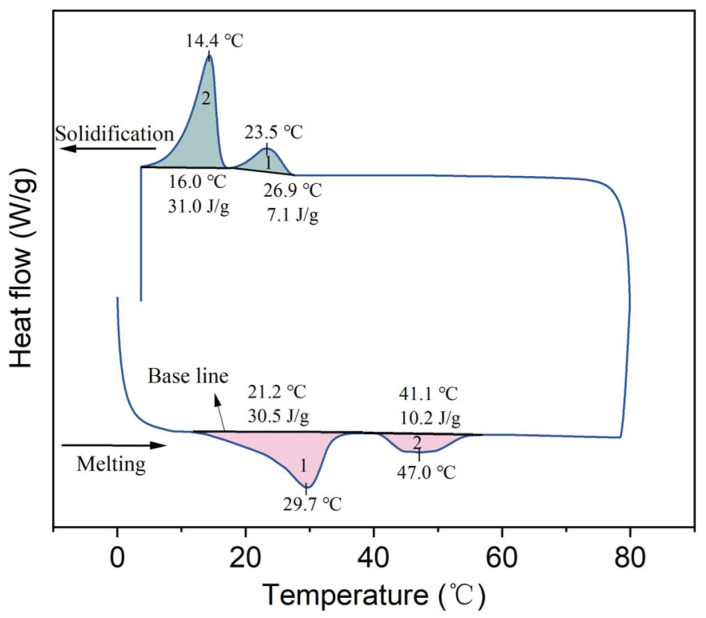
DSC curve of lard.

The DSC curve of lard is depicted in Figure 4. During the heating process, two distinct melting peaks were observed, with extrapolated onset melting temperatures at 21.2 and 41.1 °C. The first peak corresponds to the melting process of unsaturated fatty acids, such as oleic acid, while the second peak is associated with the melting process of palmitic acid. Due to the relatively low proportions of other fatty acids (i.e., lauric, myristic, and palmitoleic), there are varying degrees of deviation in the peak temperatures [22]. During the cooling process, two distinct solidification peaks were observed, and a significant degree of supercooling was detected. The specific values of the DSC curve are presented in Table 2. It is evident from the table that the phase change temperature of lard is suitable for application in building envelopes; however, further reduction in its supercooling degree is required.

### 3.2. Morphology and Structural Characteristics of Reed Straw Biochar and Bio-SSPCM

**Figure 5:**
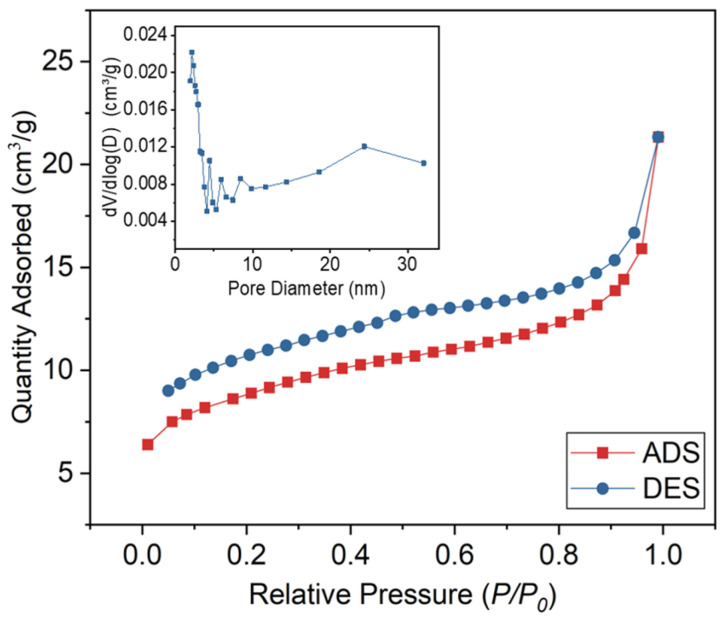
N_2_ adsorption/desorption isotherms of RSB.

The pore structures have a significant impact on the crystalline behavior and thermal stability of the composite PCMs. The N_2_ adsorption/desorption isotherm of reed straw biochar (RSB) is depicted in Figure 5, while the corresponding N_2_ physical adsorption data are presented in Table 3. According to the IUPAC adsorption isotherm, RSB can be classified as type IV isotherms. The hysteresis loop of the RSB is prominently observed within the relative pressure range of 0.11–1.0, indicating a substantial presence of pores in both the mesopore and macropore regions. RSB has hierarchical porous structure with a BET surface area of 29.58 m^2^/g, which is beneficial for PCM adsorption [23].

**Figure 6:**
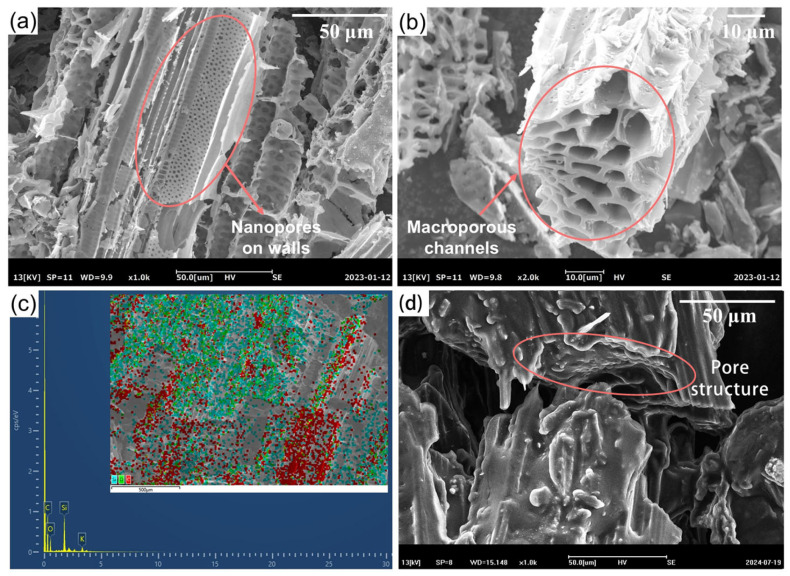
(**a**,**b**) SEM images of RSB, (**c**) EDS spectrum of RSB, and (**d**) SEM images of lard/RSB/EG.

The SEM images of reed straw biochar (RSB) are shown in Figure 6a,b. During the carbonization process, the organic compounds underwent degradation, resulting in the formation of well-defined macroporous channels with nanopores distributed inside the walls [24,25]. Figure 6c is the EDS spectrum of the RSB, which provides elemental composition information of the material. The EDS spectrum clearly shows the presence of C, O, Si, and K atoms in the RSB. Carbon (C) and oxygen (O) are the main elements, which are consistent with the organic and inorganic components of the reed straw. Silicon (Si) is likely derived from the silica in the reed straw, which is a common component in plant biomass. Potassium (K) is a mineral element present in the reed straw. The presence of these elements confirms the composition of the RSB and provides a basis for understanding its chemical properties and interactions with other components in the Bio-SSPCM. The uniform distribution of these elements, as indicated by the EDS mapping, further supports the homogeneous structure of the RSB, which is beneficial for the adsorption and stability of the lard in the composite PCM. The cross-section and vertical section of the Bio-SSPCM (lard/RSB/EG), as depicted in Figure 6d, reveal that the hierarchical porous structures are predominantly occupied by lard. The lard adheres to the channels and surface pores of the biochar, with no discernible interface between the lard and the porous biochar. The Van der Waals force and capillary effect between the lard and capillary action can effectively prevent the leakage of lard in its molten state. This hierarchical pore structure not only allows for the high loading capacity of lard but also facilitates the capillary action and Van der Waals forces between the lard and the RSB, which are crucial for preventing the leakage of lard in its molten state. The Bio-SSPCM surface also exhibits a worm-like structure of the EG, which not only serves as an adsorbent to enhance leakage resistance due to its abundant pores but also provides an excellent heat transfer pathway for improving the thermal conductivity of the Bio-SSPCM [26].

**Figure 7:**
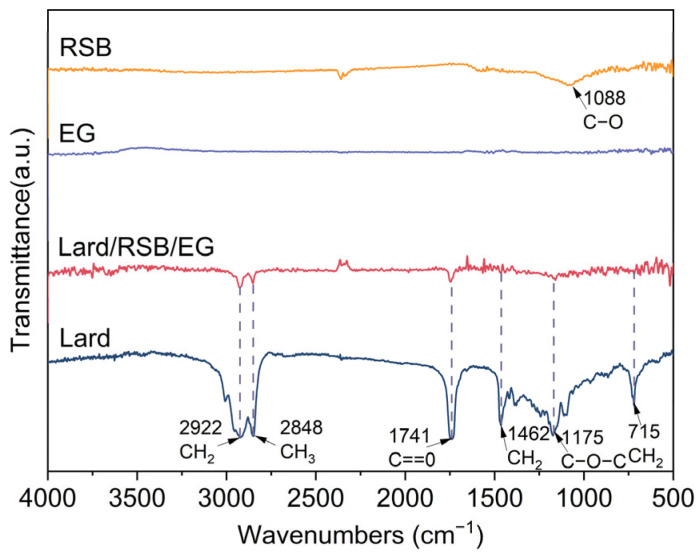
FT-IR spectra of RSB, EG, lard, and their composite, PCM lard/RSB/EG.

The FT-IR spectra of lard, EG, RSB, and lard/RSB/EG Bio-SSPCM are presented in Figure 7 to examine their chemical compatibility. The characteristic peak of RSB at 1088 cm^−1^ can be ascribed to the stretching of the siloxane group (Si-O-Si). For lard, the peaks at 2922 cm^−1^ and 2848 cm^−1^ are attributed to the asymmetric and symmetric stretching vibrations of -CH_2_ and -CH_3_ groups, respectively. The peak at 1741 cm^−1^ corresponds to the C=O stretching vibration of ester carbonyl groups, which is a typical feature of triglycerides in lard. The peaks at 1462 cm^−1^ and 1175 cm^−1^ are associated with the bending vibrations of -CH_2_ and -CH_3_ groups, and the C-O-C stretching vibration, respectively. The peak at 715 cm^−1^ is due to the rocking vibration of -CH_2_ groups, indicating the presence of long-chain alkanes in lard. In the spectrum of lard/RSB/EG Bio-SSPCM, the characteristic peaks of both lard and RSB are present, and there is no emergence or significant shift in new peaks. This indicates that there are no significant chemical interactions between lard and RSB. The physical blending of lard with RSB and EG mainly involves Van der Waals forces and physical adsorption, without breaking or forming new chemical bonds. This result is beneficial for the stability and performance of the composite PCM.

**Figure 8:**
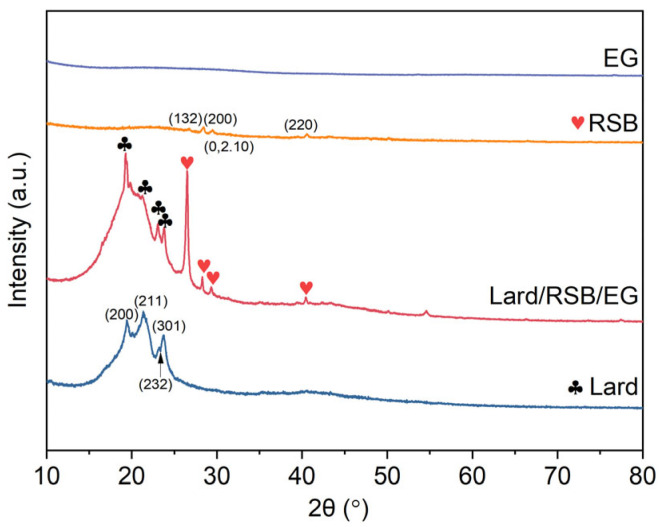
XRD patterns of RSB, EG, lard, and their composite, PCM lard/RSB/EG.

The crystalline structures of RSB, EG, lard, and their composite, PCM (lard/RSB/EG), were analyzed via XRD, as presented in Figure 8. Lard exhibited characteristic diffraction peaks at 2θ values of approximately 19.4° (200), 21.4° (211), 23.0° (232), and 23.7° (301), corresponding to its typical fatty acid triglyceride crystalline structure, which is a common feature of lipid materials. The RSB sample showed a broad peak at around 22°, indicative of an amorphous silica structure [27], consistent with the structural characteristics of biochar derived from straw biomass. Additionally, a distinct noise signal was observed near 28.4°. Notably, no new characteristic peaks were detected in the XRD pattern of lard/RSB/EG, indicating that lard retained its original fatty acid triglyceride crystalline structure and no new crystalline phases were formed. This implies that the supporting matrix (RSB and EG) primarily serves a physical supporting role, without inducing significant chemical reactions that would alter the crystalline structure of lard. Interestingly, the characteristic peak of the RSB and lard in the composite exhibited a higher level of prominence compared to that observed for RSB and lard alone. The primary reason is that the inclusion of the supporting matrix (RSB and EG) facilitates the provision of additional crystallization sites for lard, thereby promoting its crystallization process.

### 3.3. Heat Storage Properties of the Bio-SSPCM

**Figure 9:**
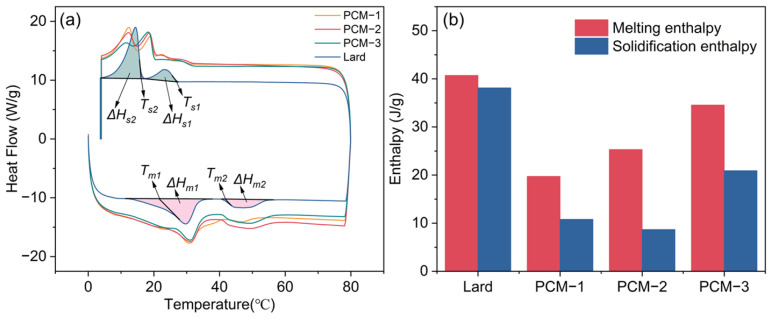
(**a**) DSC curves and (**b**) total enthalpy of the as-prepared samples.

**Figure 10:**
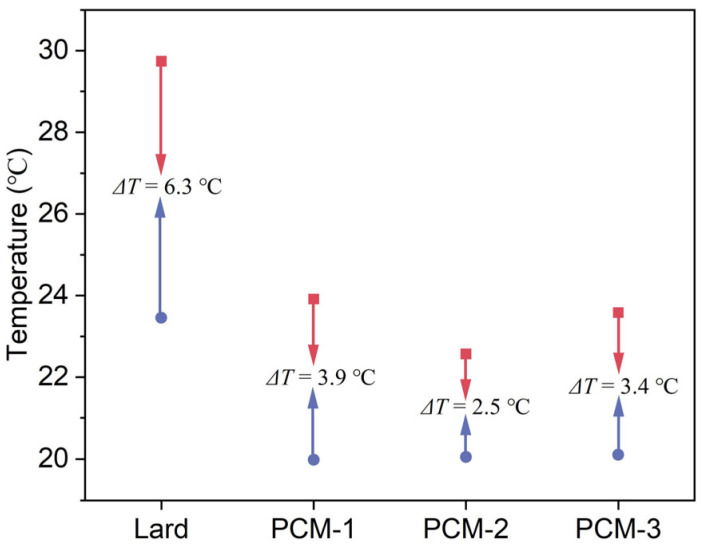
Degree of supercooling of Bio-SSPCM.

The thermal energy storage performance of the composite PCMs were determined by the differential scanning calorimetry (DSC) method and the corresponding results are presented in Figure 9 and Table 4. As Figure 9a shows, during the heating process, two endothermic peaks were observed at different melting temperatures of approximately 20 °C and 40 °C, respectively. The peak transition temperatures of Bio-SSPCM were observed to be approximately 30 °C, rendering it suitable for application in building envelopes. The total enthalpy of the Bio-SSPCM increased with the increase in lard content, as illustrated in Figure 9a. The excessive lard content, however, could result in compromised leakage resistance of Bio-SSPCM, as elucidated in the subsequent leak resistance tests. Figure 10 showed the degree of supercooling of the Bio-SSPCM. The melting peak temperature and solidification peak temperature shifted towards lower and higher temperatures, respectively. The supercooling degree of PCM-2 decreased significantly, from 6.3 °C to 2.5 °C, in comparison with pure lard. The primary reason is that the inclusion of RSB as a supporting material enables it to function as a nucleating agent, thereby alleviating the adverse effects of supercooling.

### 3.4. Leakage Resistance and Thermal Stability of the Bio-SSPCM

**Figure 11:**
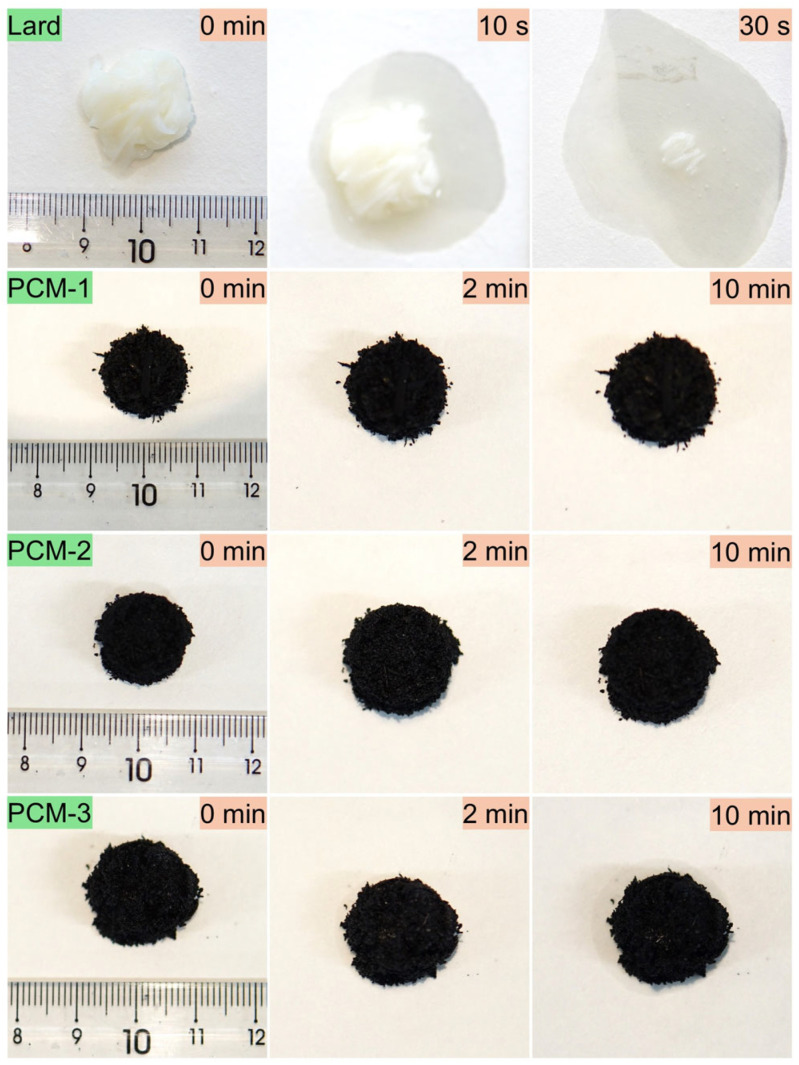
Leakage resistance testing of the as-prepared samples at 65 °C.

The leakage resistance and thermal stability of Bio-SSPCM are critical properties for practical applications. As shown in Figure 11, the prepared samples were placed on filter paper over a 65 °C constant-temperature hot plate to evaluate their leakage prevention performance. Lard completely melted within 10 min, whereas PCM-1 and PCM-2 maintained structural integrity with no visible leakage or shape deformation. Notably, lard impregnation was detected on the filter paper after heating PCM-3 for only 2 min, demonstrating its failure to meet leakage prevention requirements. In contrast, PCM-2 achieved high lard content retention without significant leakage or morphological changes, indicating its superior adsorption capacity among the tested materials.

**Figure 12:**
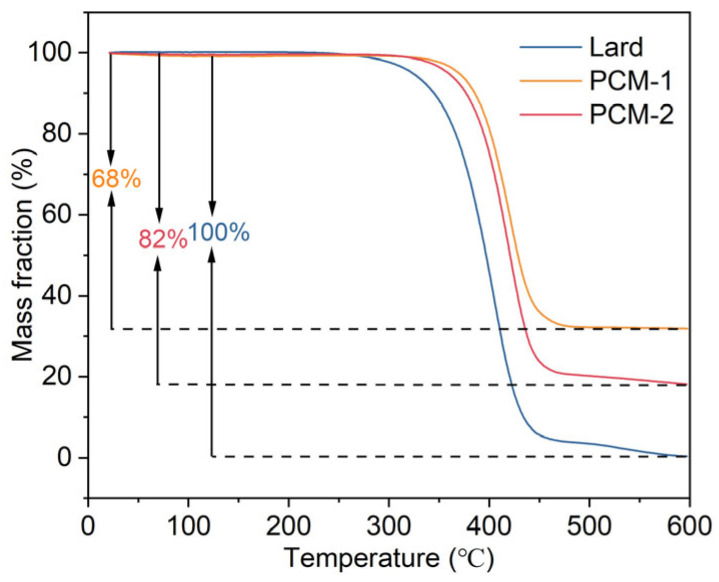
TG curves of lard, PCM-1, and PCM-2.

Thermogravimetric analysis (TGA) was utilized to investigate the decomposition behavior of Bio-SSPCM, and the results are presented in Figure 12. The degradation of lard commenced at 291 °C and was completed at 600 °C, whereas PCM-1 and PCM-2 exhibited weight loss initiation temperatures of 363 °C and 344 °C, respectively. The corresponding mass losses were measured as 68% and 82%, which correlated with the quantities of their respective compounds. These results demonstrate that RSB supporting matrix can enhance the thermal durability of the Bio-SSPCM, thereby ensuring its sufficient thermal stability for building applications.

### 3.5. Thermal Conductivity of Bio-SSPCM

**Figure 13:**
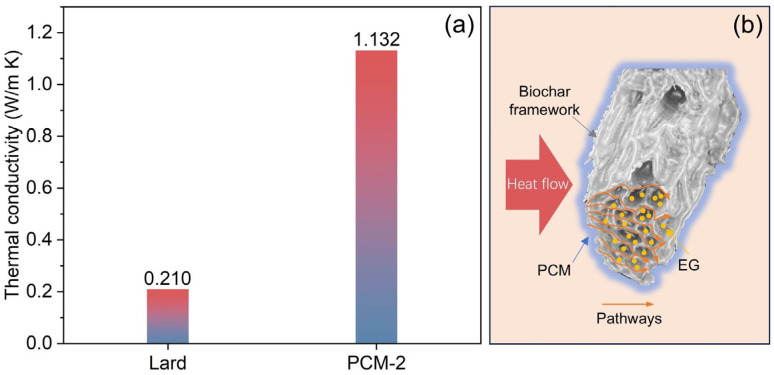
(**a**) Thermal conductivity of lard and lard/RSB/EG (PCM-2); (**b**) thermal conductivity enhancement mechanism.

The thermal conductivity analysis of bio-based PCM and dual-biomass-based shape-stabilized PCM (lard/RSB/EG) is illustrated in Figure 13. The thermal conductivity of PCM depends mainly on the matrix and collective phonon motion in a perfect crystal. Due to the lack of proper molecular alignment and atom interaction during phase change, pure PCM exhibits slower phonon motion and lower thermal conductivity [28,29]. The thermal conductivity of the Bio-SSPCM is enhanced up to 5.4 times with the addition of RSB and EG. The first reason is the addition of reed straw biochar as a supporting matrix can provide a heat transfer pathway for phonon motion in a particular direction over long distance [28]. The secondary contributing factor lies in expanded graphite’s (EG) ability to occupy interstitial spaces within the three-dimensional biomass framework. This structural integration not only strengthens the interfacial adhesion between the components of Bio-SSPCM but also establishes continuous thermal pathways, thereby enhancing the composite’s overall thermal conductivity. The mechanism illustrating the enhancement of thermal conductivity is depicted in Figure 13b.

### 3.6. Melting and Solidifying Process of Bio-SSPCM

**Figure 14:**
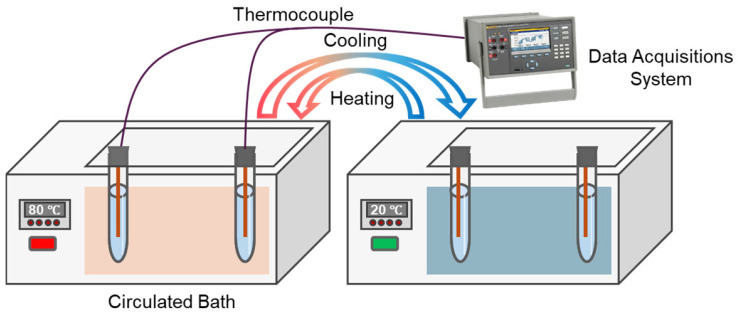
Materials melting and solidification test device.

**Figure 15:**
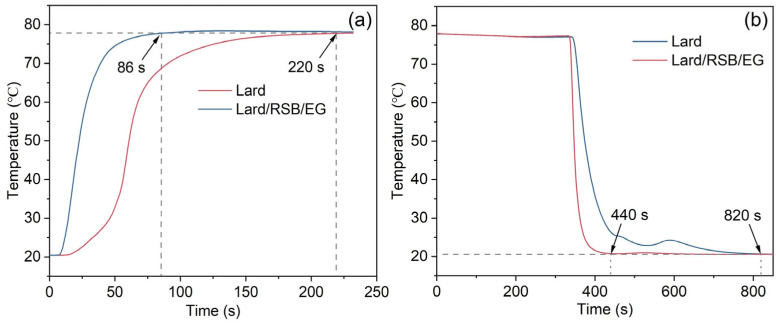
(**a**) Melting curves of lard and PCM-2; (**b**) cooling curves of lard and PCM-2.

The thermal regulation performance of Bio-SSPCM was investigated using a customized test system(Figure 14), as described in our previous study [12], and the results are shown in Figure 15. In brief, two glass tubes containing lard and PCM-2 were used as samples and subjected to a melting process by immersing them in a water bath at 80 °C. Subsequently, the solidification process was initiated by placing these glass tubes in water at 20 °C. The melting process of lard from 20 °C to 78 °C took 220 s, while PCM-2 only required 86 s, resulting in a reduction of 60.9%, as Figure 15a shows. Similarly, the solidification time for lard during the cooling process was 820 s, whereas PCM-2 only required 440 s for solidification, resulting in a reduction of 46.3%, as depicted in Figure 15b. The experimental results demonstrate a significant enhancement in the thermal response of the prepared Bio-SSPCM, enabling rapid energy storage and release during the phase transition process.

### 3.7. Thermal Reliability of Bio-SSPCM

**Figure 16:**
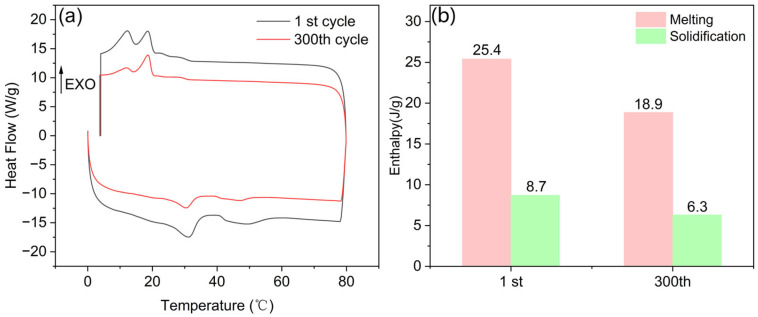
(**a**) DSC curves of PCM-2 after 300 thermal cycles; (**b**) enthalpy of PCM-2 during 300 thermal cycles.

Finally, to assess the thermal reliability of PMM-2, heating and cooling cycle experiments were carried out using differential scanning calorimetry (DSC), and the results are depicted in Figure 16. As illustrated in Figure 16a, the melting and solidification peaks from two representative cycles are highly consistent in both position and shape, indicating that PCM-2 exhibited favorable thermal cycling stability. Figure 16b displays the melting and solidification enthalpy values of PCM-2 in the first and 300th cycles. After 300 thermal cycles, although a certain degree of enthalpy reduction was observed, the overall heat storage capacity remained at a relatively high level, further confirming the excellent thermal reliability of PCM-2.

## 4. Conclusions

The present study utilized lard derived from waste fat as an environmentally sustainable organic PCM, which was combined with biochar obtained from waste reed straw to create a dual-biomass-based shape-stabilized PCM (Bio-SSPCM) for efficient thermal energy storage. The thermal conductivity of the Bio-SSPCM was improved by incorporating expanded graphite (EG), and the mechanism behind this enhancement in heat conduction was revealed, leading to the following conclusions:

(1) FT-IR and GC-MS analysis of lard showed that the lard extracted from pork contains four types of fatty acids: 38.03% oleic acid (unsaturated acid), 12.73% palmitic acid, 21.49% stearic acid, 12.09% hexadecanamide, and other fatty acids. Due to the liquid state of unsaturated fatty acids at room temperature, the lard exhibits a gel-like structure at room temperature. DSC test results show that waste fat has the phase transition temperatures about 20–40 °C and a melting enthalpy value of 40 J/g, which is suitable for building passive cooling applications.
(2) The prepared reed straw biochar (RSB) has hierarchical porous structure with a BET surface area of 29.58 m^2^/g, which is beneficial for PCM adsorption. When combined with lard, it can accommodate up to 80% loading without leakage. The peak transition temperatures of Bio-SSPCM were observed to be approximately 30 °C, with a melting enthalpy of 25.3 J/g, making it suitable for application in building envelopes.
(3) The thermal conductivity of the Bio-SSPCM (lard/RSB/EG) is enhanced up to 5.4 times with the addition of RSB and EG. The first reason for this is that the addition of reed straw biochar as a supporting matrix can provide a heat transfer pathway for phonon motion in a particular direction over long distance. The second reason is that the infiltration of EG into the pores of the biochar framework enhances the carbon content in the Bio-SSPCM, which directly contributes to the observed increase in thermal conductivity.

## Figures and Tables

**Table 1:** The compositions of the Bio-SSPCM.

Samples of Bio-SSPCM	Lard (g)	Reed Straw (g)	EG (g)
PCM-1	7.0 ± 0.1	2.1 ± 0.1	0.4 ± 0.01
PCM-2	8.0 ± 0.1	1.6 ± 0.1	0.4 ± 0.01
PCM-3	9.0 ± 0.1	0.6 ± 0.01	0.4 ± 0.01

**Table 2:** DSC results of lard.

Peaks	Melting Process			Solidification Process		
	*T_m_* (°C)	*T_peak-m_* (°C)	Δ*H_m_* (J/g)	*T_s_* (°C)	*T_peak-s_* (°C)	Δ*H_s_* (J/g)
Peak 1	21.2	29.7	30.5 ± 1.2	26.5	23.5	7.1 ± 0.4
Peak 2	41.1	47.0	10.2 ± 0.5	16.0	14.4	31.0 ± 1.3
Total			40.7 ± 1.5			38.1 ± 1.5

**Table 3:** Textural properties of RSB.

BET Surface Area (m^2^/g)	Adsorption Total Pore Volume (cm^3^/g)	Average Pore Diameter (nm)
29.58 ± 0.89	0.0329 ± 0.001	4.46 ± 0.15

**Table 4:** DSC results of Bio-SSPCM.

Samples	Melting Process				Solidification Process			
	*T_m_*_1_ (°C)	Δ*H_m_*_1_ (J/g)	*T_m_*_2_ (°C)	Δ*H_m_*_2_ (J/g)	*T_s_*_1_ (°C)	Δ*H_s_*_1_ (J/g)	*T_s_*_2_ (°C)	Δ*H_s_*_2_ (J/g)
PCM 1	23.9	18.6 ± 0.3	42.9	1.1 ± 0.1	19.9	3.1 ± 0.1	14.2	7.7 ± 0.2
PCM 2	22.6	18.4 ± 0.2	40.9	7.0 ± 0.3	20.1	2.8 ± 0.1	14.3	5.9 ± 0.1
PCM 3	23.6	26.2 ± 0.4	41.0	8.3 ± 0.4	20.1	20.9 ± 0.3	-	-

## Data Availability

The original contributions presented in this study are included in the article. Further inquiries can be directed to the corresponding authors.
